# Deep lymphaticovenular anastomosis for refractory lymphorrhea following thigh sarcoma resection: A case report

**DOI:** 10.1016/j.jpra.2026.07.034

**Published:** 2026-07-26

**Authors:** Yuma Togashi, Hisashi Sakuma, Takako Fujii, Eri Matoba, Megumi Ooida

**Affiliations:** a Department of Plastic and Reconstructive Surgery, Ichikawa General Hospital, International University of Health and Welfare, Ichikawa, Japan; b Department of Plastic and Reconstructive Surgery, Keio University School of Medicine, Tokyo, Japan

**Keywords:** Lymphaticovenular anastomosis, Deep lymphatic vessel, Refractory lymphorrhea, Soft tissue sarcoma, Two–compartment lymphoscintigraphy

## Abstract

Lymphorrhea is a common complication following wide resection of soft tissue sarcomas of the thigh. When deep lymphatic vessels are disrupted, conservative management alone may be insufficient. Lymphaticovenular anastomosis (LVA) targeting superficial lymphatic vessels has been reported as treatment for refractory lymphorrhea, while deep LVA (d–LVA) has been reported mainly for established lymphedema. Here we extend this approach to refractory postoperative lymphorrhea. We report a 53–year–old man who underwent wide resection of a left medial thigh myxoid liposarcoma with femoral artery and vein resection, followed by femoral artery reconstruction using a contralateral small saphenous vein graft and soft tissue coverage with a pedicled rectus abdominis musculocutaneous flap. He developed profuse refractory lymphorrhea and lower extremity edema. Initial LVA targeting superficial lymphatic vessels at two sites did not improve drainage, and a concurrently placed free omental graft failed and was removed. Two–compartment lymphoscintigraphy demonstrated leakage from the deep lymphatic system near the femoral canal resection margin. On postoperative day 48, additional LVA was performed at three sites, targeting deep lymphatic vessels alongside the posterior tibial vessels together with nearby superficial lymphatic vessels, and para–SSV lymphatic vessels that communicate with the deep system through the deep fascia at the popliteal fossa. Drain output decreased from approximately 200 to 19 mL/day within seven days, and lymphoscintigraphy confirmed complete resolution of leakage. No recurrence was observed within 94 days. These findings suggest that D–LVA may be effective in managing refractory lymphorrhea, and that comprehensive evaluation of the deep lymphatic system using two–compartment lymphoscintigraphy may facilitate accurate and efficient surgical planning.

## Introduction

Lymphorrhea is a common complication following wide resection of soft tissue sarcomas of the thigh.^1^^,^^2^ Prolonged lymphorrhea increases the risk of wound dehiscence, surgical site infection, delayed adjuvant therapy, and chronic lymphedema.^3^

Conservative measures such as drainage and compression therapy are usually sufficient. However, when deep lymphatic vessels are disrupted, conservative treatment alone may be insufficient. Several reports have demonstrated the efficacy of lymphaticovenular anastomosis (LVA) targeting superficial lymphatic vessels in refractory lymphorrhea.^4^, ^5^, ^6^ Deep LVA (d–LVA) has been reported for established lymphedema; here we explore its application to refractory postoperative lower extremity lymphorrhea, in which the goal is rapid control of an active leak by lymphovenous bypass rather than long–term decongestion. We report a case of refractory lymphorrhea following thigh sarcoma resection successfully managed with two–compartment lymphoscintigraphy–guided combined superficial and deep LVA.

## Case report

A 53–year–old man underwent wide resection of a left medial thigh myxoid liposarcoma with femoral artery and vein resection. The femoral artery was reconstructed using a contralateral small saphenous vein graft, with soft tissue coverage by a pedicled rectus abdominis musculocutaneous flap.

**Fig. 1:**
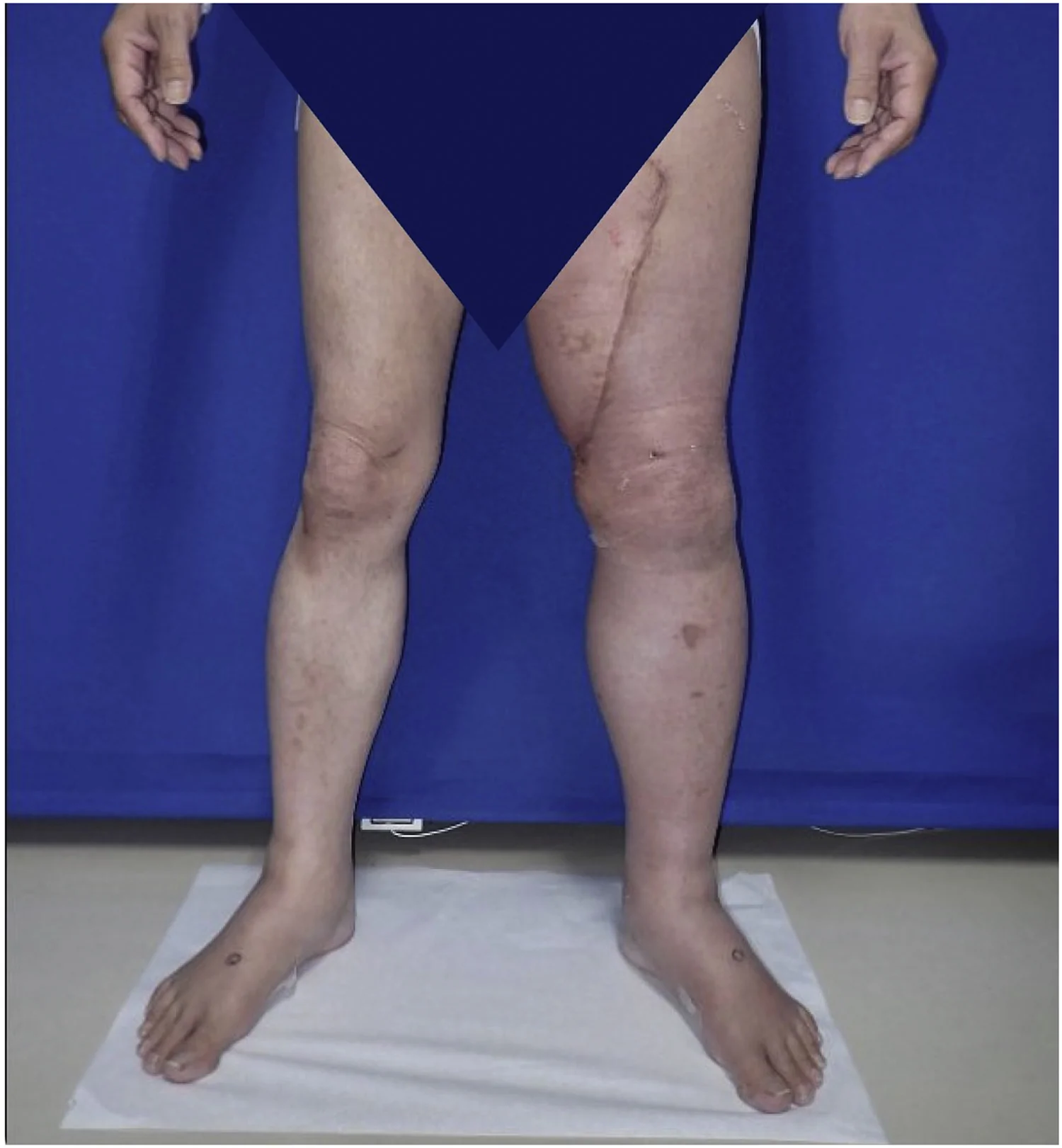
Clinical photograph of the lower extremities, showing marked edema of the left limb associated with refractory lymphorrhea following wide resection of a left thigh myxoid liposarcoma.

**Fig. 2:**
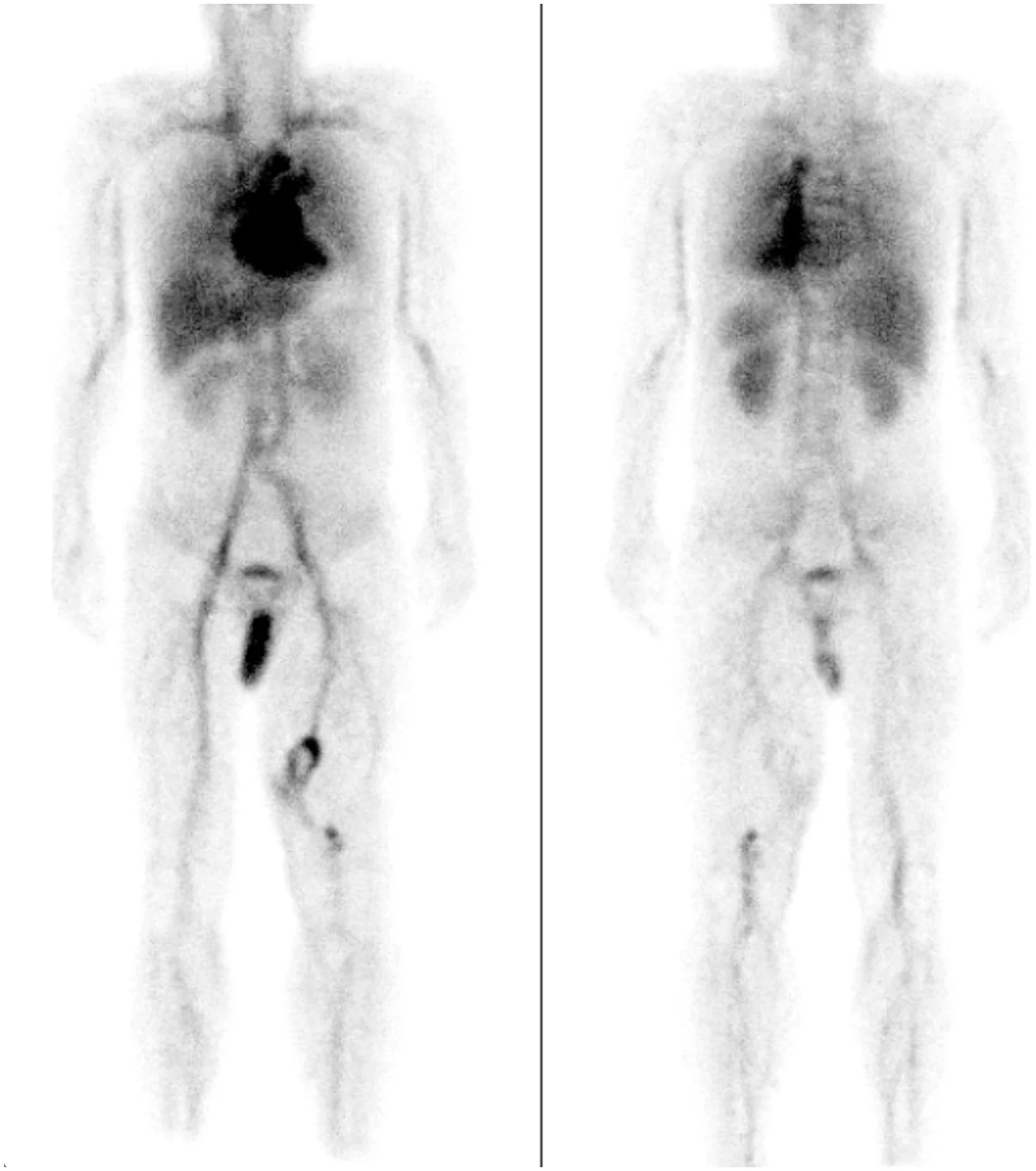
Two–compartment preoperative lymphoscintigraphy. (A) Deep–system imaging following subfascial radiotracer injection, showing focal accumulation at the resection margin along the femoral canal, indicating leakage from the deep lymphatic system. (B) Superficial–system imaging following subcutaneous radiotracer injection, showing normal lymphatic flow along the medial lower extremity without abnormal accumulation.

**Fig. 3:**
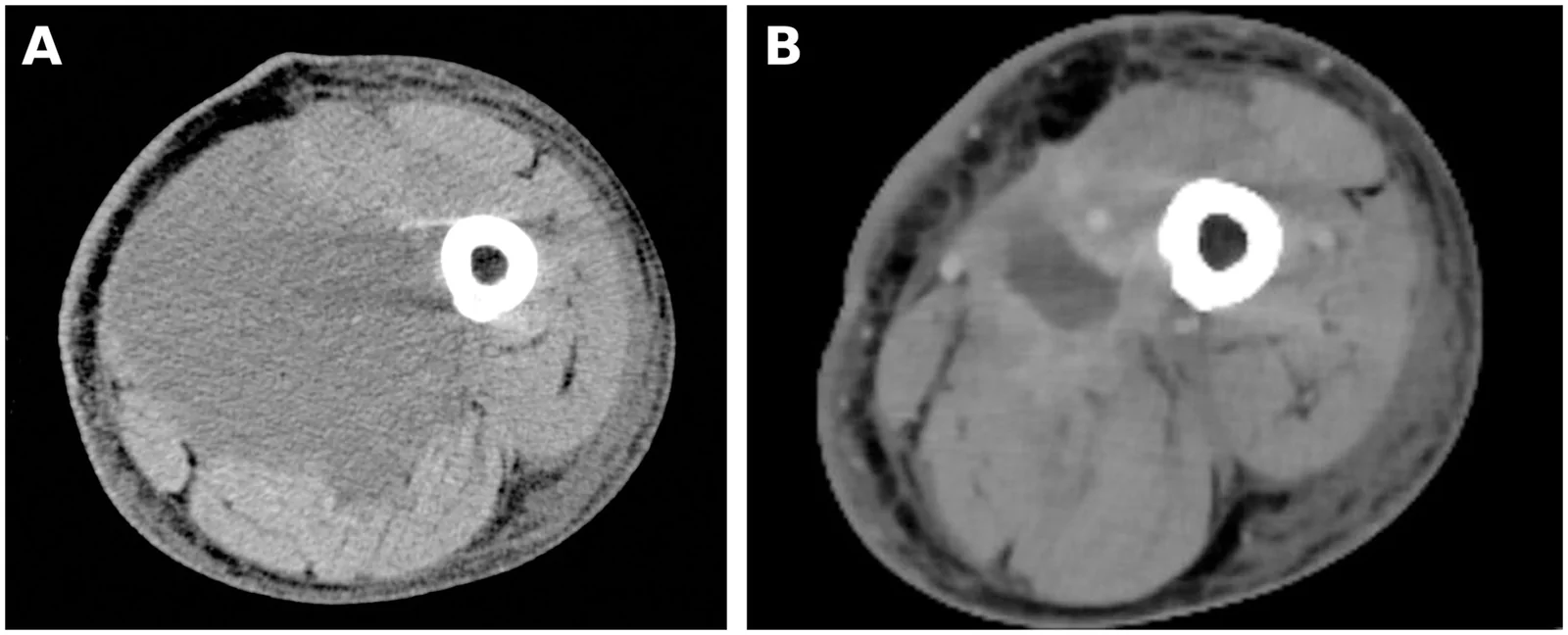
Axial CT comparison of the left thigh before and after D–LVA. (A) Preoperative CT showing diffuse lymphatic fluid accumulation with poor delineation of muscle planes. (B) Postoperative CT showing marked reduction in fluid and clear delineation of muscle compartments.

Postoperatively, the patient developed profuse lymphorrhea (Fig. 1) refractory to drainage and compression therapy. Two–compartment lymphoscintigraphy separately evaluates the superficial and deep lymphatic systems via subcutaneous and subfascial radiotracer injections, respectively, with the subfascial injection performed under ultrasound guidance beneath the plantar aponeurosis.^7^ This examination was performed on postoperative days 21 and 23, revealing no superficial lymphatic leakage (Fig. 2B) but significant deep lymphatic leakage near the resection margin along the femoral canal (Fig. 2A). On postoperative day 26, free omental transfer was attempted concurrently with LVA targeting superficial lymphatic vessels at two sites in the medial infrapatellar and medial malleolar regions. However, omental graft perfusion deteriorated, necessitating its removal on postoperative day 29. Lymphorrhea persisted, with the drain output remaining approximately 200 mL/day (Fig. 3A).

Because superficial LVA was insufficient, D–LVA was planned under local anesthesia on postoperative day 48. Preoperative lymphoscintigraphy and ultrasonography identified deep lymphatic vessels alongside the posterior tibial vessels together with nearby superficial lymphatic vessels in the same field, and para–SSV lymphatic vessels that communicate with the deep system through the deep fascia at the popliteal fossa. The small saphenous vein (SSV) served as the anatomical landmark for the para–SSV lymphatic vessels. Incision sites were planned accordingly.

**Fig. 4:**
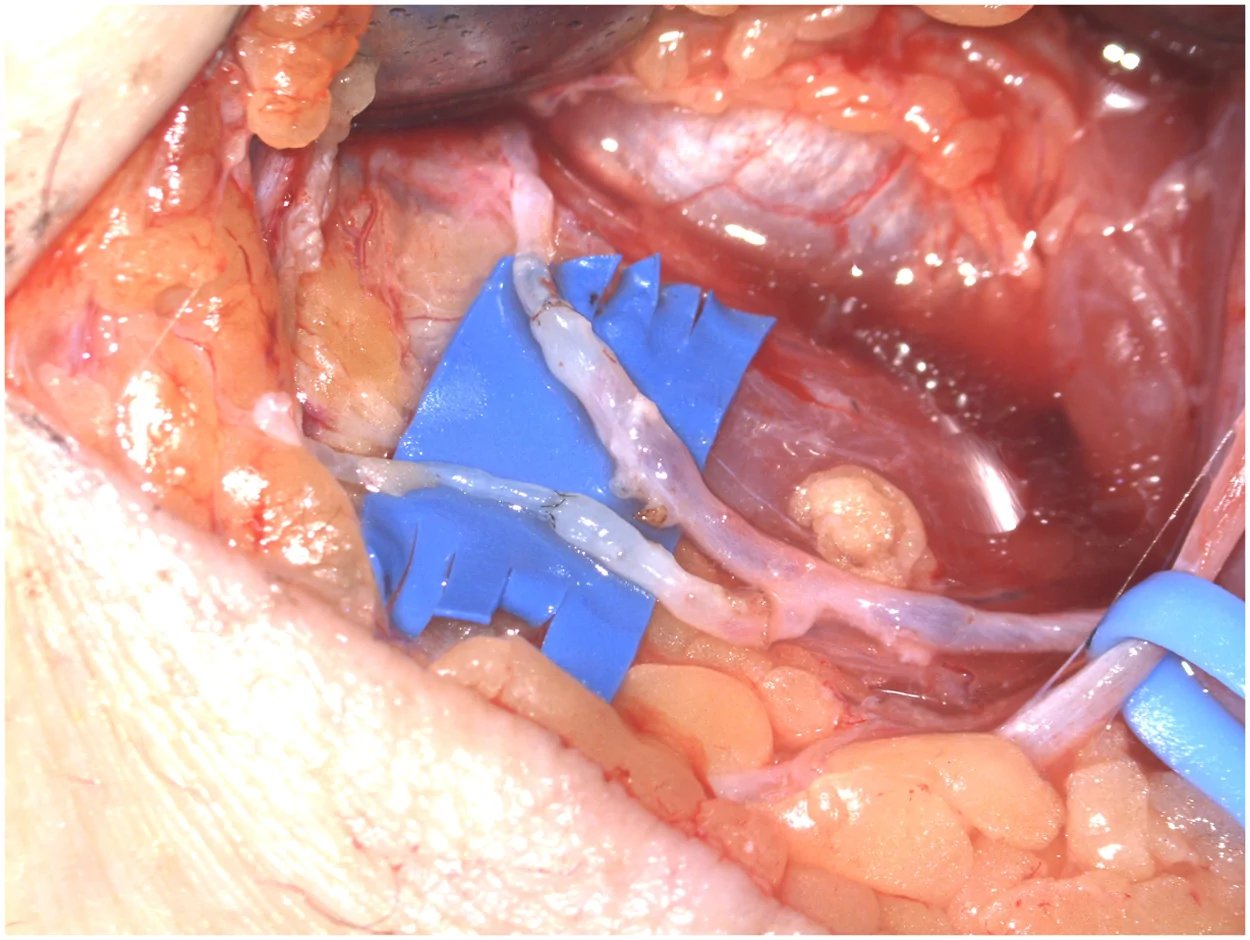
Intraoperative photograph at the distal medial leg showing deep lymphatic vessels (white) exposed beneath the deep fascia alongside the posterior tibial vascular bundle. A blue silicone background sheet enhances visualization of the lymphatic vessels; the blue vessel loop on the right encircles the recipient venous structure.

Surgery was performed through two incisions at the distal medial leg and the popliteal fossa (Fig. 4). At the distal medial leg, deep lymphatic vessels along the posterior tibial vessels and superficial lymphatic vessels were each anastomosed to branches of the great saphenous vein (GSV). At the popliteal fossa, a para–SSV lymphatic vessel was anastomosed to a superficial vein. All three anastomoses were end–to–end with 12–0 nylon interrupted sutures under an operating microscope; vessel diameters were 0.4–0.6 mm. Patency was confirmed by intraoperative venous filling after clamp release. ICG lymphography was not used, as the deep lymphatic vessels could not be visualized by superficial dermal injection.

Drain output markedly decreased from postoperative day 1, declining from approximately 200 to 19 mL/day by day 7 after D–LVA. CT showed substantial reduction in subcutaneous edema (Fig. 3B). Lymphoscintigraphy on postoperative day 62 demonstrated complete resolution of leakage. No recurrence of lymphorrhea or lymphedema was observed within 94 days of follow–up.

## Discussion

Lower extremity lymphorrhea is predominantly iatrogenic, occurring in up to 55% of patients following resection of malignant soft tissue tumors of the thigh.^1^ The risk is particularly high for anteromedial tumors, as their resection may necessitate sacrifice of the femoral and adjacent deep lymphatic vessels.^2^

LVA is an effective alternative when conservative treatment fails. Giacalone et al. achieved complete resolution in all 13 patients with recurrent lymphocele or lymphorrhea using ICG–guided LVA.^4^ Yang et al. reported resolution or marked improvement in 8 of 10 patients with lymphorrhea treated by superficial LVA.^5^ Kadota et al. required approximately seven anastomoses per patient for pelvic/inguinal lymphorrhea with superficial LVA.^6^ Superficial approaches thus tend to require multiple anastomoses. In contrast, the present case was resolved with only three anastomoses, suggesting that targeting the deep system may reduce the number required.

Deep LVA (d–LVA) has been reported for lymphedema. Yamamoto et al. treated postmastectomy upper extremity lymphedema by anastomosing deep lymphatic vessels along the brachial artery to adjacent venules.^8^ Scaglioni et al. demonstrated that the addition of D–LVA to superficial LVA in patients with persistent lymphedema yielded further therapeutic improvement.^9^ These pioneering reports addressed established lymphedema. The present case extends D–LVA to refractory postoperative lymphorrhea, where the primary objective is rapid control of leakage by lymphovenous bypass. Vascularised lymph node and lymph vessel transfer (VLNT/VLVT), established for chronic lymphedema, act over longer time scales and may not control an active deep–lymphatic leak as rapidly as microsurgical anastomosis.

Pan et al. reported that deep lymphatic vessels run along the anterior tibial, posterior tibial, and peroneal vascular bundles, converging at the deep popliteal nodes.^10^ The superficial and deep systems communicate through perforating vessels penetrating the deep fascia. Superficial vessels along the SSV drain into the superficial popliteal nodes and transition into the deep system at the popliteal fossa via these perforators. Deep lymphatic flow then ascends along the femoral vessels to the inguinal nodes.

In the present case, leakage originated from the deep system where vessels along the femoral canal had been transected. The initial superficial LVA could not address this source, underscoring the value of evaluating the deep system when superficial LVA fails.

In our patient, rapid improvement was achieved with only three anastomoses through two small incisions under local anesthesia, suggesting that identification of responsible deep vessels by two–compartment lymphoscintigraphy supports efficient surgical planning and can guide the choice between superficial LVA alone and D–LVA.

Preventive strategies, such as preoperative ICG lymphography to identify and preserve at–risk lymphatic channels and immediate LVA at the time of tumor resection, have been proposed. However, in the present case the responsible vessels lay in the deep subfascial layer and would not have been detected by superficial dermal ICG injection. Moreover, the failure of subsequent superficial LVA to reduce drain output suggests that immediate superficial LVA alone might have offered limited preventive benefit when the deep system is the principal source. Two–compartment lymphoscintigraphy may therefore complement these strategies by selecting patients requiring deep–system intervention.

Several limitations should be acknowledged. The 94–day follow–up is insufficient to assess long–term lymphedema, although the lymphorrhea resolved with disappearance of leakage on lymphoscintigraphy. Multiple sequential interventions including superficial LVA, omental flap, and continuous drainage preceded D–LVA, so their contribution cannot be excluded. However, the abrupt and sustained decrease in drain output occurring only after D–LVA, together with disappearance of leakage on lymphoscintigraphy, suggests that D–LVA was the principal contributing factor. The findings should be considered suggestive rather than conclusive. In addition, D–LVA was combined with concurrent superficial LVA, so the specific contribution of D–LVA alone cannot be isolated. Lymphoscintigraphic interpretation was qualitative; quantitative analysis was not performed. Further cases with longer follow–up are needed to define indications for D–LVA in postoperative lymphorrhea.

## Conclusion

In this case, D–LVA targeting subfascial and perforating lymphatic vessels was suggested to be effective for refractory lower extremity lymphorrhea. Two–compartment lymphoscintigraphy supported anatomically guided planning, which may allow effective treatment with fewer anastomoses than superficial LVA alone. Further cases are needed to establish indications.

## Authors’ contributions

Yuma Togashi: Conceptualization, Investigation, Writing - original draft, Writing-review & editing, Visualization. Hisashi Sakuma: Conceptualization, Investigation, Supervision, Writing - review & editing. Takako Fujii: Investigation, Writing - review & editing. Eri Matoba: Investigation, Writing - review & editing. Megumi Ooida: Investigation, Writing - review & editing.

## Informed consent and patient details

Written informed consent was obtained from the patient for publication of this case report and accompanying images.

## Declaration of generative AI and AI–assisted technologies in the manuscript preparation process

During the preparation of this work, the authors used Claude (Anthropic) for English language proofreading and editing assistance. After using this tool, the authors reviewed and edited the content as needed and take full responsibility for the content of the published article.

## Funding

This study received no specific grants from any funding agency in the public, commercial, or not–for–profit sectors.

## Declaration of competing interest

The authors declare that they have no conflicts of interest.

## Data Availability

The data that support the findings of this study are not publicly available due to patient privacy concerns but are available from the corresponding author upon reasonable request.

## Appendix Supplementary materials

Image, application 1

Image, application 2

Image, application 3
