# Supplementary material for: Deep lymphaticovenular anastomosis for refractory lymphorrhea following thigh sarcoma resection: A case report
Source: JPRAS Open. 2026 Jul 26;51:703–9. doi: 10.1016/j.jpra.2026.07.034 (PMC13506793; doi:10.1016/j.jpra.2026.07.034)
Supplement: Supplementary file 1 [file mmc1.docx]

SDC1_paraSSV_LVA
